# Investigation of the Guinea fowl and domestic fowl hybrids as potential surrogate hosts for avian cryopreservation programmes

**DOI:** 10.1038/s41598-019-50763-3

**Published:** 2019-10-03

**Authors:** Mariann Molnár, Bence Lázár, Nikoletta Sztán, Barbara Végi, Árpád Drobnyák, Roland Tóth, Krisztina Liptói, Miklós Marosán, Elen Gócza, Sunil Nandi, Michael J. McGrew, Eszter Patakiné Várkonyi

**Affiliations:** 1 National Centre for Biodiversity and Gene Conservation, Institute for Farm Animal Gene Conservation, Gödöllő, Hungary; 2 NARIC Agricultural Biotechnology Institute, Department of Animal Biotechnology, Applied Embryology and Stem Cell Research Group, Gödöllő, Hungary; 3 University of Veterinary Medicine, Department of Exotic Animal and Wildlife Medicine, Budapest, Hungary; 4 The Roslin Institute and Royal Dick School of Veterinary Studies, University of Edinburgh, Easter Bush Campus, Midlothian, EH25 9RG UK

**Keywords:** Stem-cell biotechnology, Agricultural genetics, Genetic hybridization, Embryonic germ cells

## Abstract

In the last decade, avian gene preservation research has focused on the use of the early precursors of the reproductive cells, the primordial germ cells (PGCs). This is because avian PGCs have a unique migration route through the vascular system which offers easy accessibility. Furthermore, culturing of the cells *in vitro*, freezing/thawing, reintegration into a recipient embryo and the development of the germ cells can be carried out in well-defined laboratory circumstances. The efficient recovery of the donor genotype and the frequency of germline transmission from the surrogate host animals are still areas which need further development. Thus, the aim of the present study was to investigate an infertile interspecific hybrid (recipient) as an appropriate host for primordial germ cells from native poultry breeds. Guinea fowl × chicken hybrids were produced, the crossing was repeated inversely. The phenotype, the hatching time, the hatching rate, the sex ratio, the presence of own germ cells, the fertility and the phenotype of viable hybrids and the incidence of chromosomal abnormalities of dead hybrid embryos were described. 6.65% viable offspring was obtained with crossing of Guinea fowl females with domestic fowl males. Crossing of domestic fowl hens with Guinea fowl male resulted in lower fertility, 0.14% viable offspring. Based on the investigations, the observed offspring from the successful crossing were sterile male hybrids, thus an extreme form of Haldane’s rule was manifested. The sterile hybrid male embryos were tested by injecting fluorescently labeled chicken PGCs. The integration rate of labeled PGCs was measured in 7.5-day, 14.5-day and 18.5-day old embryonic gonads. 50%, 5.3% and 2.4% of the injected hybrid embryos survived and 40%, 5.3% and 2.4% of the examined gonads contained fluorescent labeled donor PGCs. Therefore, these sterile hybrid males may be suitable recipients for male PGCs and possibly for female PGCs although with lower efficiency. This research work shows that the sterility of hybrids can be used in gene conservation to be a universal host for PGCs of different avian species.

## Introduction

According to the International Union for Conservation of Nature and Natural Resources 14% of avian species are listed as threatened with extinction^1^. Therefore, it is extremely important to develop integrated systems for avian germplasm conservation; however, techniques established for genetic conservation for mammals cannot be transferred directly to avian species, mainly because of the unique physiological and anatomical characteristics of the egg^2^.

Nowadays avian genetic conservation is primarily focused on the *in situ* approach: maintaining live collections of birds. This strategy is expensive and carries the risk of infectious diseases, environmental disasters and loss of genetic variability. Therefore, the conservation process should be supported by an *ex situ* strategy^3–5^ as well. Such a strategy includes the cryopreservation of embryos, gametes or different types of embryonic cells to be stored in a gene bank for future demand and subsequently used to recreate the species of interest after recovery of the frozen reproductive material. Primordial germ cells or PGCs are the only cells in the developing embryo which can transmit the genetic information to the next generation. These cells are the precursors of adult germ cells and among the embryonic stem-like cells in the bird embryo. PGCs have unique characteristics and accessibility. They migrate through the vascular system to colonize the developing gonads; therefore they can easily be isolated from and reintegrated into the embryonic circulatory system^6^ with the goal of production of germline chimeras. These germline chimeras, ‘surrogate hosts’, can be bred and will give rise to a progeny carrying the genome of the donor PGCs. PGCs have been successfully used for regenerating individual purebred chicken from frozen germplasm^7–9^.

**Figure 1:**
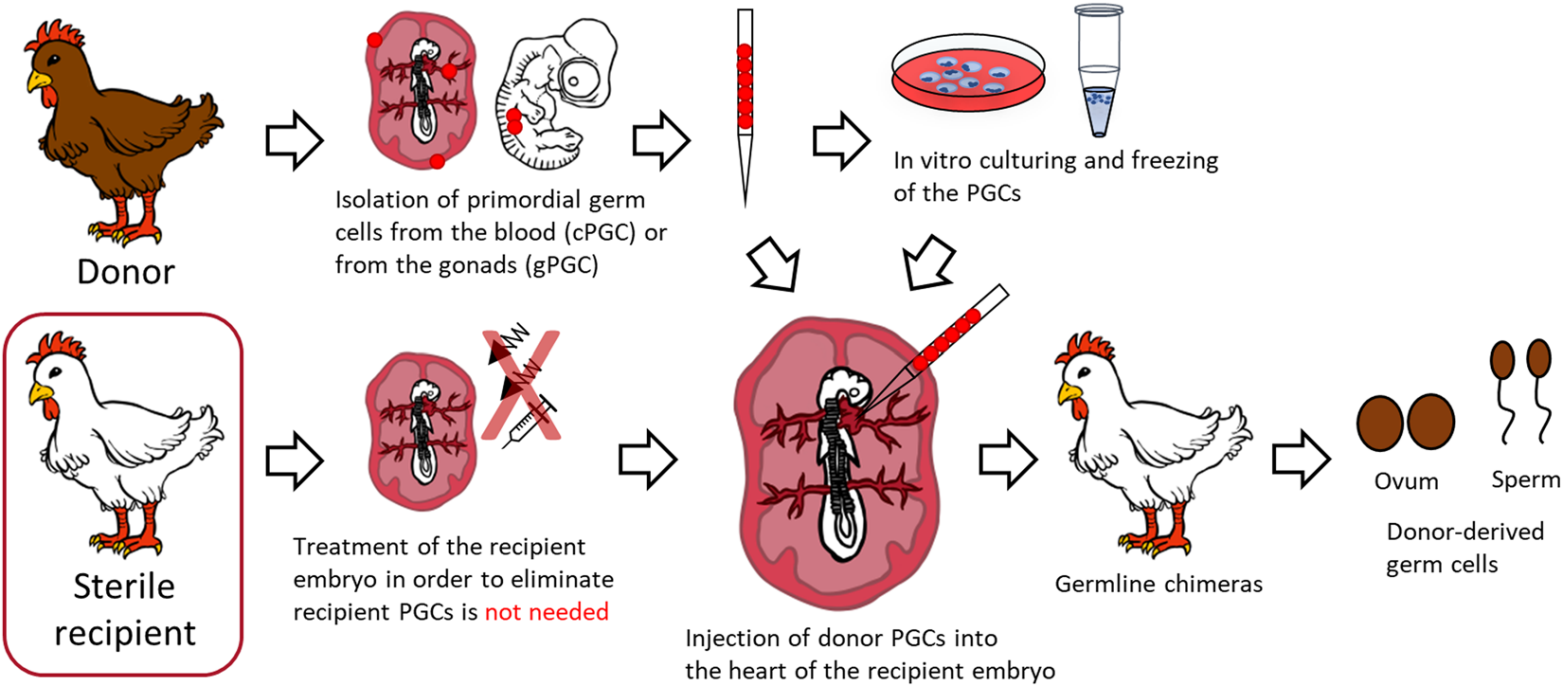
General outline of producing donor-derived hatchlings with sterile recipients. The migration of PGCs reaches its peak in the bloodstream between HH stages 13–17 (48–65 hours after laying in chicken); thus this is the optimal stage for collecting donor PGCs, and also this is the suitable stage for injecting them back to the recipient embryo. After the isolation, using a selective media, *in vitro* cultures of PGCs will be possible. For long term storage we cryopreserved the cells and keep them in liquid nitrogen. As a next step, cryopreserved or fresh PG cells are injected into the recipient embryo. After the hatching the presumptive germline chimeras are crossed back with the original breed or with each other to regenerate the donor genotype. With the usage of sterile hybrids, the treatment of the recipient embryos is not needed therefore the process is more efficient.

To apply this method, it is essential to use recipient host embryos (Fig. 1). Since the gonad of the recipient embryo contains its own germ cells, the offspring will be of dual origin. Therefore, to enhance the efficiency of the colonization and transmission of the exogenous PGCs, it is desirable to lower the number of endogenous PGCs in the host. The first successful experiments aiming this were based on UV^7^ or χ-radiation^10–14^. Using these techniques, more donor-derived PGCs colonized the recipient gonads, but the development of the treated embryos fell behind the non-treated control embryos^10^.

Due to the limitations in large-scale application and embryo development, researchers started to develop more precise methods for targeting the PGCs and not the entire embryo. Busulfan is an alkylating agent^15–17^ which was previously used in mammals for sterilization and causes irreversible damage to the germ cells of the recipient, then after 10 hours decomposes rendering it suitable for germ cell transplantation. In birds, a similar effect was observed^8,18,19^, but distributing the chemical in a lipid filled environment was a challenge to be met. Researchers developed a technique during which the busulfan is delivered directly to the embryo by injection of an emulsion^20^. The effectiveness of this method was a significant improvement; donor cells colonized the recipient gonads with 99.5% success and germline transmission of donor PGCs was improved^21^. Recently, precision genome editing techniques are also proven to be useful in this field. Taylor *et al*.^22^ used transcription activator-like effector nucleases (TALEN)-mediated gene targeting to produce sterile female chicken recipients for PGC germline transmission studies. Although gene editing for sterility has further possibilities creating suitable recipients, there are many countries with strict genetically modified organisms (GMO) policy and some cases even complete prohibition of GMO animals. Therefore, we chosen an alternative approach to produce sterile recipients through hybrid sterility by crossing domestic fowl with Guinea fowl. As previously described in numerous studies, hybrids between different species are often sterile dependent on the phylogenetic distance between them.

Postzygotic incompatibility induced hybrid sterility and inviability and their evolutionary aspects have been studied in *Drosophila*^23^, frogs^24^, butterflies^25^ and birds^26–28^. An increase of postzygotic developmental isolation between species correlated with divergence time was shown in these studies.

A first review of avian hybrid literature was undertaken by Suchetet in 1897^29^. Later, many more compilations of avian hybrids were published^30–37^. Interspecific hybrids can be observed among several species of Pheasant (*Phasianidae*) and Guinea fowl (*Numididae*) families due to their genetic similarity^38^. Hanebrink was the first, who reported on the characteristics and behaviour of naturally occurring hybrids between Guinea fowl and domesticated chicken^39^. Hanebrink^40^ also reported on naturally occurring hybrids between Guinea fowl and peafowl. Domesticated chicken and Japanese quail were crossed successfully by Mitsumoto and Nishida^41^ and by Wilcox and Clark^42^. Warren and Scott^43^ reported on domesticated chicken-turkey hybrids. According to other publications, only few fertile eggs and few advanced embryos could be obtained by this combination^44–47^. According to Ghigi^48^, domestic fowl-Guinea fowl and Guinea fowl-peafowl crosses resulted only male offsprings. Cole and Hollander^49^ reported that only male offsprings hatched by crossing of male pigeon and female dove, however, the crossing with swapped sexes generated both male and female offspring. Domesticated chicken with Guinea fowl and domesticated chicken with Japanese quail was crossed by Mathis and McDougald^50^ in order to investigate the resistance of hybrids to host specific *Eimeria* infections. Tubaro and Lijtmajer^51^ reported successful interspecific crosses in relation of duck species. According to this research, with growing phylogenetic distance the reproductive isolation increases between hybridizing species. In case of sympatric species, the reproductive isolation is greater than in case of allopatric species with the same level of divergence, furthermore, hybrid crosses conform to Haldane’s rule. Out of the 161 successful crossings, in 125 cases the males outnumbered females, 23 were equal to the sex ratio, and in 13 cases the proportion of females was higher among the offsprings than the males^51^.

The aim of the present study was to establish the chicken × Guinea fowl as an appropriate interspecific hybrid (recipient) for receiving primordial germ cells (PGCs) from indigenous poultry breeds and according to our expectations, the donor genotype may appear among the offsprings of the hybrids (Fig. 1). Guinea fowl × chicken hybrids were produced, the crossing was repeated inversely. The developmental phenotype, the hatching time, the hatching rate, the sex ratio, the fertility of viable hybrids, presence of own germ cells and the phenotype and the incidence of chromosomal abnormalities of dead hybrid embryos are described and the possible causes of infertility were also investigated.

## Methods

### Ethics statement

Animals were kept and maintained according to general animal welfare prescriptions of the Hungarian Animal Protection Law (1998; XXVIII). Permission to undertake experimental animal research at the National Centre for Biodiversity and Gene Conservation was granted by the National Food Chain Safety Office, Animal Health and Animal Welfare Directorate, Budapest (Permission No. PE/EA/2485-6/2016). All experimental methods described herein were approved by the Institutional Ethics Review Board of the Institute for Farm Animal Gene Conservation (No. 7/2011).

### Experimental design of interspecific crossings

**Figure 2:**
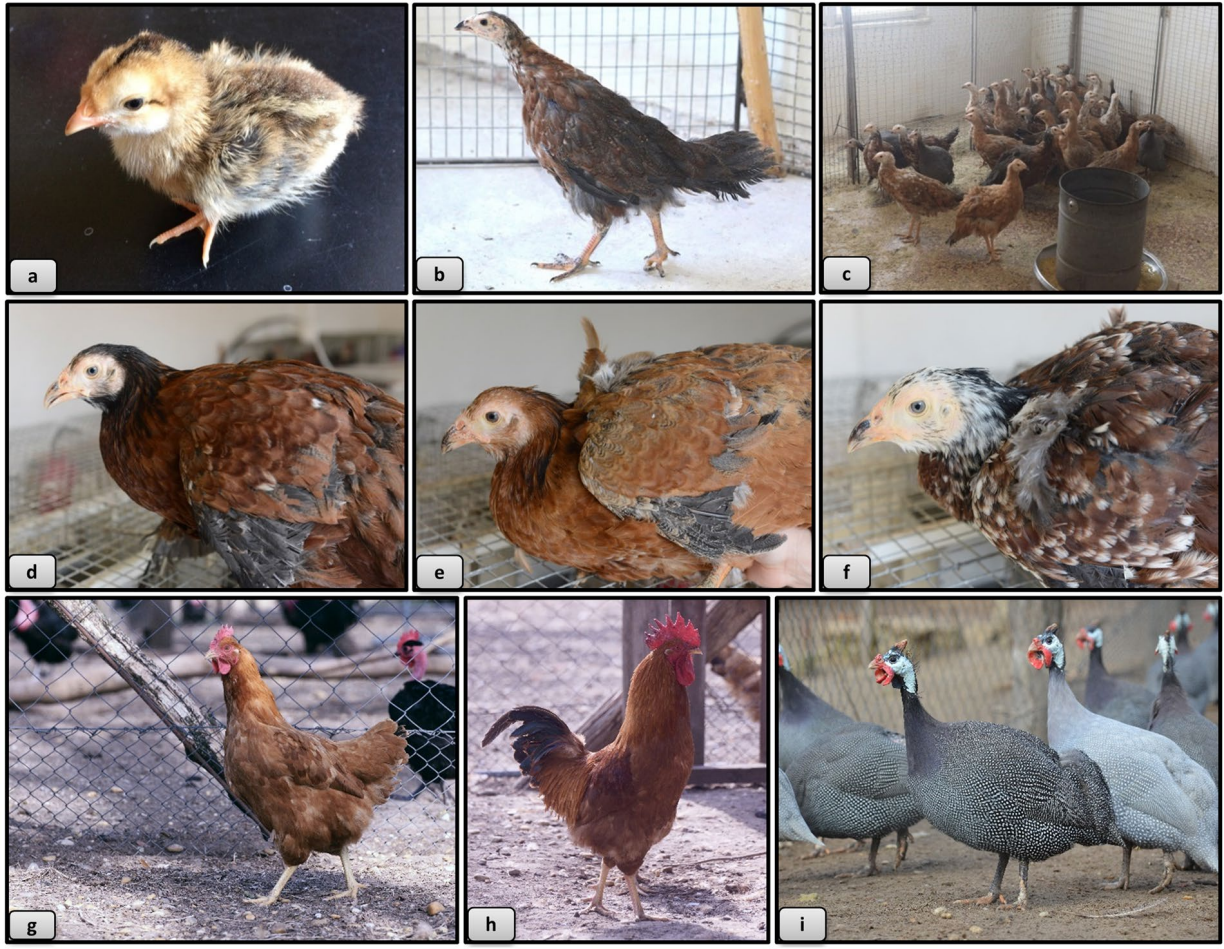
Phenotype of hybrids, Yellow Hungarian chicken and Guinea fowl. **(a)** 1 day old hybrid, (**b)** 8 weeks old hybrid, (**c)** 16 weeks old hybrids, (**d)** brownish colour variety, (**e)** yellow colour variety, (**f**) white, mixed colour variety, (**g)** Yellow Hungarian hen, (**h)** Yellow Hungarian rooster, (**i)** Guinea fowls.

In the first year of the experiment 60 Guinea fowl females were artificially inseminated with mixed sperm of 20 Hungarian yellow cockerels (Fig. 2h).

In the second year of the experiment 50 Hungarian yellow hens (Fig. 2g) were artificially inseminated with mixed sperm of 30 Guinea fowl males (Fig. 2i).

Eggs were collected twice a day, and then placed into an incubator for hatching every 10 days. In the first year 972, in the second year 701 eggs were placed into the hatching machine in two incubation cycles.

### Characteristics of the species and varieties involved in the crossing experiments

#### Hungarian landrace guinea fowl

Guinea fowl (Fig. 2i) is considered as an adapted Hungarian poultry species. There are three color variants in Hungary: bluish-gray, white and the original wild color. It has excellent meat quality, very good ability to adapt to different conditions, resistance, wild and seeking habit and low maintenance costs^38^. Body weight of the male is 1.30 to 1.60 kg, while that of the female is 1.20 to 1.40 kg. It starts laying eggs at the end of April and 50 to 80 very hard-shell eggs are laid every year. The hatching time of Guinea fowl is 28 day. The chicklings are yellowish brown with longitudinal darker bands on the back at hatching^38^.

#### Yellow hungarian chicken breed

Yellow Hungarian Chicken breeds (Fig. 2g,h) belong to the medium size, dual-purpose breeds. Hens weigh 2.0 to 2.3 kg, while cockerels weigh 2.5 to 3.0 kg^38^. The highest value of these chicken breeds of a fine bone structure was their fine-fibred, excellent and palatable meat, which generated a demand for them in domestic and foreign markets alike. Their annual egg production reached 140 to 150 eggs, based on which it was assigned as an excellent dual-purpose breed. The hatching time is 21 day. The chicks at hatching have pure yellow color^38^.

### Maintenance of Guinea fowl and domestic fowl experimental stocks

The Hungarian Landrace Guinea fowl and Yellow Hungarian chicken breed used in this study were kept at the National Centre for Biodiversity and Gene Conservation, Institute for Farm Animal Gene Conservation (Gödöllő, Hungary). The specimens used in the experiment were kept in individual cages with light program (14 L: 10D). Eggs were collected twice a day and stored at 15–17 °C. The birds were fed *ad libitum* with granulated laying mash in addition to continuous water supply.

### Artificial insemination

Semen from both cockerels and Guinea fowl was collected by abdominal massage according to Burrows and Quinn^52^. The fresh, pooled and diluted semen was inseminated in a dose of 100 ± 20 million spermatozoa/female in all cases. For the calculation of sperm concentration Lake’s diluent was used.

### Incubation of the eggs and investigation of embryo survival and the phenotypic analysis of dead embryos

Incubation was carried out with a MIDI F500S hatchery machine (PL Machine Ltd., Tárnok, Hungary) with two 45° rotations per hour. The incubation temperature was 37.8 °C, relative humidity 70%.

Egg fertility was determined by candling at 8^th^ and 14^th^ day after egg set. Apparent clear eggs and eggs with abnormal embryos were opened and examined. Any embryonic malformations found were recorded. Eggs detected as apparently infertile at candling, sometimes contained very early dead embryos or embryonic tissue. Early dead embryos or embryonic tissues were removed and put into 0.9% NaCl solution for phenotype classification under a dissecting microscope (Olympus). They were classified according to Abbot and Yee^53^ modified by Szalay^54^, while very early embryonic death (which occurred before laying) stages were classified according to Eyal-Giladi and Kochav^55^. The following phenotype categories^56^ were determined:

- No development (ND): Infertile egg.
- Positive development - (PD): The sheets of membranes consist of ectodermal and endodermal tissue only. Blood vessels are not developed.
- Blastoderm without embryo - (BWE): Ectodermal, endodermal and also mesodermal tissue can be observed. Blood islets are formed.
- Dead embryo - (for example D1–5): The embryos died at various stages of development during the five day incubation period.
- Abnormal embryo - (AE): Living embryos showing any malformations or retarded growth.

### Cytogenetic analysis of dead embryos

For cytogenetic studies, embryonic tissues were transferred into 0.56% KCl (Reanal, No. 822930) solution containing 10 µl of Vinblastine (Sigma, V-1377) for mitotic arrest and they were incubated for 20 minutes in 37.5 °C (Memmert, BE 200). Finally they were fixed with several changes of fixative (acetic acid:absolute ethanol - 1:3). Slides were prepared from fixed tissues suspended in 50% acetic acid. Karyotypes were analyzed after Giemsa staining under microscope (Zeiss Axioskop 2 plus) at magnification x1250.

### Maintenance of the hatched viable hybrid offspring

The hatched hybrids were grown in a special chick rearing box until 4 weeks of age (0.5 m^2^/10 individuals). The box is equipped with automatic heating and lighting. The temperature was gradually reduced weekly from 30 °C to the final 22–24 °C. After 4 weeks of age they were placed on deep litter. The young individuals were fed *ad libitum* with granulated starter mash (Szinbád Ltd., Gödöllő, Hungary) and water supply.

### Verifying hybrid status with molecular genetic markers

There are Japanese quail microsatellite markers which amplify genomic DNA in both Guinea fowl and chicken species^57^. 15 Japanese quail markers were tested on control chicken and Guinea fowl samples. GUJ1 and GUJ87 amplified a DNA product in both species of different allele sizes, making it possible to detect a chicken-Guinea fowl hybrid using these markers. Blood samples were taken from 48 sacrificed adult offsprings (38 of them deemed to be hybrids and 11 control Guinea fowl) for DNA extraction. DNA samples were isolated using a salting-out method^58^ modified for poultry species. The control DNA samples used for the fragment analysis were randomly selected from 12 chicken and Guinea fowl individuals.

For the polymerase chain reaction (PCR), tailed primers^59^ were used with different fluorescent labels. GUJ1 was labeled with the WellRED dye D4 (forward: 5′-CAGGACCAGGCTACCGTGGAAGCGAAAGCCGAGCCA-3′; reverse: 5′-CAGCACTTCGGAGCACAGGA-3′) and the primer GUJ87 was labeled with the WellRED dye D3 (forward: 5′-CGGAGAGCCGAGAGGTGCATGCCGGCTGCTATGACAG-3′; reverse: 5′-AAGTGCAGGGAGCGAGGAAG-3′). The master mix contained 5 µM of each primer, 20 mM MgCl2 (10x Dream Taq Buffer, Thermo Fisher Scientific), 25 mM dNTP mix (Thermo Fisher Scientific) and 5U/µL Taq DNA polymerase (Dream Taq DNA polymerase, Thermo Fisher Scientific). The cycling parameters were as follows: 95 °C for 4 min., followed by 35 cycles of 95 °C for 15 s, 58 °C for 30 s and 72 °C for 1 min, with a final step at 72 °C for 9 min and then hold at 10 °C (SuperCycler Trinity, Kyratec).

The fragment analysis was performed with capillary gel electrophoresis by Beckman Coulter automatic DNA sequenator (GenomeLab GeXP). In our case, three different WellRED dyes (recommended by the manufacturer’s instructions) were used for the microsatellite detection, D4 (blue) for GUJ1, D3 (green) for GUJ87 and D1 (red) for the size standard. Genotyping data were analyzed with the GenomeLab GeXP System fragment analysis software (Beckman Coulter) that identified the allele sizes for the microsatellite markers of both species.

### Immunostaining of PGCs

The hybrid eggs were placed into the incubator. In case of the chimaeras, the injection of GFP-PGCs was performed on day 3 and then the eggs were sealed and put back into the incubator. On the 10th, 18.5th and 20th day of embryonic development, the eggshell was removed. The embryonic gonads were dissected, and the tissue was fixed in 4% formaldehyde.

#### Immunostaining of PGCs in the 10- and 20-day old hybrid embryos

The tissue was equilibrated in 15% sucrose, embedded in 7.5% gelatin/15% sucrose, and frozen rapidly in isopentane on dry ice. Sections were cut at 10 µ thickness in a cryostat, and mounted on glass slides (Superfrost). Gelatin was removed from the slides in PBS at 38 °C. Slides were heated to 121 °C in an antigen retrieval solution (Access Supreme, Menarini Diagnostics). Sections were washed in PBS + 0.05% Tween (PBST), followed by 5 minutes in PBST + 0.5% Triton X, then washed again in PBST before blocking in 5% goat serum in PBST. Slides were incubated with antibodies diluted 1:250 in blocking agent, overnight at 4 °C. Consecutive sections were incubated with antibodies against either p63 (Abcam ab124762) or SSEA-1 (mouse anti-SSEA-1, 1:10, Developmental Studies Hybridoma Bank, US). Sections were washed in PBST and incubated for 1 hour with secondary antibodies Alexa Fluor 488-conjugated goat anti-rabbit IgG (ThermoFisher, A-11034) or Alexa Fluor 546-conjugated goat anti-mouse IgM (ThermoFisher, A-21045), stained with Hoechst 33342 (10 µg/mL) and visualized with a fluorescence microscope (Leica DMLB).

#### Immunostaining of PGCs in the 18.5-day hybrid chimaera gonads

After fixation, the tissue was equilibrated in 10%, 20% and then 30% sucrose, embedded in 7.5% gelatin and frozen rapidly in isopentane on liquid nitrogen. Sections were cut at 10 µ thickness in a cryostat and mounted on glass slides. Gelatin was removed from the slides in 1x PBS at 38 °C. After washing with PBS, the slides were blocked and permeabilised with 0.1% BSA-PBS containing 2.5% donkey serum and 0.1% Triton X-100 (Merck Millipore, US) for 45 minutes at room temperature. Then, slides were incubated overnight with rabbit antiVASA (1:1000; kindly provided by Bertrand Pain, Lyon, France) primary antibody in a humid chamber at 4 °C. As a next step, the slides were washed three times in 1x PBS. Incubation in the secondary antibody followed: donkey anti-rabbit IgG conjugated to Alexa 555 (1:400, Molecular Probes Inc., USA) in a dark humid chamber for 1 hour at room temperature. After washing once with 1x PBS, the nucleus was stained with TO-PRO®-3 stain (1:500, Molecular Probes Inc., US), which is a far-red fluorescent (642/661) nuclear and chromosome counterstain. After another 3 rounds of 1x PBS wash coverslips were mounted on the slide with the application of 20 μl VECTASHIELD® Mounting Media (Vector Laboratories Inc., US) and analysed by confocal microscopy (TCS SP8, Leica). Negative controls were stained only with the secondary antibody.

### Histological analysis of gonads of the raised hybrids

38 hatched hybrid individuals and 11 Guinea fowl controls were raised to maturity and in every two weeks between the 16^th^ and 30^th^ week of growth 4–5 hybrids and one or two control animals were sacrificed for histological analysis. The gonads were removed, imaged, and then fixed in 8% paraformaldehyde solution for 1–2 days (Excelsior AS Tissue Processor, No.: A82300001, Thermofisher).

Gonads were washed under running water, dehydrated in increasing concentrations of ethanol, then transferred to paraffin at 75 °C and placed in an embedding cassette (Paraffin Dispenser WD-4C, No.: 205510, Kunz Instrumentz) for the preparation of histological sections. Paraffin was congealed on a cooling plate (Cooling Plate CP-4D, No.: 205600, Kunz Instrumentz) and the paraffin blocks were cut into 3–4 µm-thick sections. After the hardening of the sections, hematoxylin-eosin staining was performed (Shandon Varistain 24–4 Slide Stainer, No.: 8358-30-1025) and the sections were covered for microscopic examination (Zeiss Axioskop 2 plus) at x100 magnification.

### Injection of GFP-expressing PGC lines into 3-day-old hybrid embryos

In order to test the functional and structural integrity of the hybrid gonads, GFP-expressing PGCs were injected into 3-day-old hybrid embryos. The GFP-expressing PGC lines were isolated from transgenic White Leghorn chicken embryos (the GFP expressing White Leghorn line was established by McGrew *et al*.^60^). PGCs were cultured *in vitro* using a specific media with 0.2% chicken serum and method described by Whyte *et al*.^61^. A male GFP expressing cell line (4ZP) was selected for injection^62^. Hybrid eggs were incubated to stage HH15-16. 5000–10000 PGCs, in 1 µl of culture media were injected into the heart of each hybrid embryo through a hole (5–6 mm in diameter) on the eggshell. After the injection, 50 µl of sterile 1x D-PBS was added and then 2 layers of parafilm were used to close the hole^14^. The injected eggs were incubated at 37.8 C with 70% relative humidity. Later on, the embryos were sacrificed at day 7.5, day 14.5 and day 18.5 to screen the gonads for the integrated GFP-expressing PGCs. The gonads were dissected from the embryos and were emended in ProLong Gold antifade reagent (Ref.: P36934 Molecular Probes, USA) then imaged using a fluorescent stereomicroscope (Leica M205 FCA, Leica Ltd., Germany).

### Perivitelline sperm penetration assay (PSPA)

Because of poor fertility of eggs of Hungarian Yellow’s hens inseminated with Guinea fowl semen, perivitelline sperm penetration assay (PSPA) was used to check the sperm penetration in freshly laid eggs^63^. 80 eggs were stored at 16 °C before assessment. After opening the eggs, the separated yolks were placed in physiological salt solution. A 1 × 1 cm piece of the perivitelline layer from over the germinal disc was cut around and washed in 0.9% physiological salt solution. The yolk- and albumen-free piece of membrane was spread on a microscope slide. The hydrolyzed points produced by spermatozoa in the inner perivitelline layer over the germinal disc (IPVL holes) were viewed with a ×4 objective using dark field optics, and the total number of IPVL holes from over the germinal disc was counted manually^64^.

### Statistical analysis

Statistical analysis and figures were made using R Studio (version 1.2.1335), R (version R-3.2.2.) and package “ggplot2” (H. Wickham. ggplot2: Elegant Graphics for Data Analysis. Springer-Verlag New York, 2016.) Pairwise comparisons (Chi-squared tests) between pairs of proportions with correction for multiple testing (“Holm” correction method) were used to compare the four experimental groups (two hybrid and two control groups) in case of infertility, embryonic death, abnormality and hatching (p < 0.05 was considered significant).

## Results

### Description of the phenotype of hatched hybrids

One week old hybrids were phenotypically different from the monochromatic light brown Hungarian yellow chicks. Similarly to guinea fowl chicks, the hybrids had black stripes on their back. On the 18^th^ week, hybrids were bigger in stature than both of the parent species, their back was slightly curved, and they had long downward tails (Fig. 2).

Basically, three different phenotypes were observed in the hybrids: a dark brown (Fig. 2d), a light brown (Fig. 2e) and a white spotted type (Fig. 2f), but none of the hybrids had helmet, crest or facial wattles which distinguished them from pure Guinea fowl. They also had a feather free face and a strong, slightly curved beak (Fig. 2).

### Hatching time, fertility and phenotypic analysis of dead embryos for different hybrid matings

#### Guinea fowl hen × Hungarian Yellow cockerel

**Figure 3:**
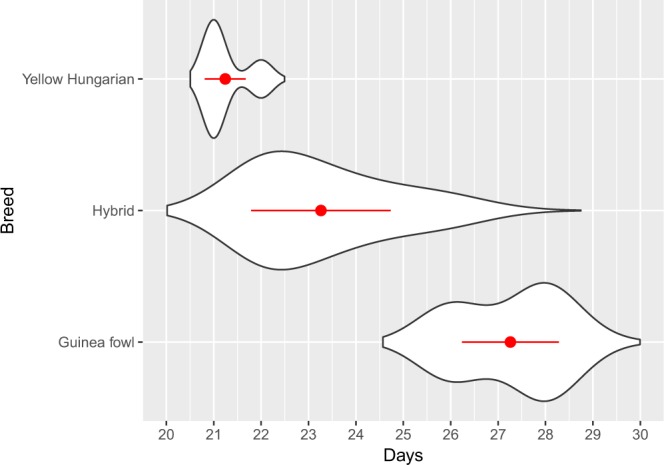
Hatching time of the hybrid eggs in comparison with the original breeds. The hatching time of hybrid, Yellow Hungarian and Guinea fowl eggs were monitored. The eggs of the Yellow Hungarian chicken and the Guinea fowl hatched after 21–22 days and 26–29 days, respectively. In case of the hybrid eggs, an extended period of time was experienced (from day 21 to 27) which is a transition between the Yellow Hungarian and the Guinea fowl. Mean values and standard deviations are shown with red colour.

For control inseminations of chicken hens using cockerel semen; 54.6 percent of chicken hens were fertilized. Following artificial insemination of Guinea fowl hens, 31.7% of the incubated eggs were infertile which is significantly better than the opposite hybrid crossing (Hungarian Yellow hen × Guinea fowl male), similar to the Guinea fowl control, but worse than the chicken control (Table 1). For the remaining eggs, 29.6% of the incubated eggs underwent an early embryonic death (5.98% PD and 23.61% BWE) and the number of dead embryos (D1–5) was 13.3% within 5 days after the start of incubation. There was no significant difference in the number of PD embryos between the Guinea fowl hen × Hungarian Yellow cockerel hybrid and the control groups, but there were significantly more BWE embryos among the hybrids. Following this, the number of dead hybrids deceased with developmental stages; 1.66% between days 6 and 10 of incubation (D6–10), 1.2% between day 11 and 15 (D11–15), 2.1% between the days 16 and 20 (D16–20), 2.1% between 21 and 27 days (D21–27). The number of dead embryos increased perinatally to 10.75% which we attribute to the inability to pip through the extremely hard eggshell. In case of embryonic death (D1–5, D6–10, D11–15, D16–20, D21–27), abnormality and perinatal death the proportion of hybrid embryos did not differ significantly from either of the control groups (Table 1). The remaining 6.7% hatched normally (Table 1). Hatching of healthy hybrids occurred between 21 to 27 days of incubation (Fig. 3). 0.9% of the dead embryos had obvious abnormalities such as closure of cranial bones, abdominal wall defects, beak deformities and dwarfism.

**Table 1:** The number of hatchlings and eggs affected by infertility or early embryonic death in two hybrid matings and the control groups.

	ND	PD	BWE	D1-5	D6-10	D11-15	D16-20	D21-27	AE	Perinatal death	Hatched	Ʃ (No.)
Guinea fowl × chicken hybrid	286 (31.7%)^A^	54 (5.98%)^A^	213 (23.61%)^A^	120 (13.3%)^A^	15 (1.66%)^A^	11 (1.21%)^A^	19 (2.1%)^A^	19 (2.1%)^A^	8 (0.88%)^A^	97 (10.75%)^A^	60 (6.65%)^A^	902
Chicken × Guinea fowl hybrid	691 (98.43%)^B^	6 (0.85%)^B^	2 (0.28%)^B^	0 (0.0%)^B^	0 (0.0%)^B^	0 (0.0%)^B^	1 (0.14%)^B^	0 (0.0%)^B^	0 (0.0%)^A^	1 (0.14%)^B^	1 (0.14%)^B^	702
Chicken control	23 (16.31%)^C^	8 (5.67%)^A^	1 (0.7%)^BC^	11 (7.8%)^A^	4 (2.83%)^A^	0 (0.0%)^AB^	4 (2.83%)^A^	0 (0.0%)^AB^	0 (0.0%)^A^	13 (9.21%)^A^	77 (54.6%)^C^	141
Guinea fowl control	20 (26.31%)^AC^	7 (9.21%)^A^	3 (3.94%)^C^	7 (9.21%)^A^	3 (3.94%)^A^	0 (0.0%)^AB^	0 (0.0%)^AB^	0 (0.0%)^AB^	0 (0.0%)^A^	9 (11.84%)^A^	27 (35.52%)^D^	76

ND: No development; PD: Positive development; BWE: Blastoderm without embryo; D1–5: Died on day 1 to 5 of incubation; D6–10: Died on day 6 to 10 of incubation; D11–15: Died on day 11 to 15 of incubation; D16–20: Died on day 16 to 20 of incubation; D21–27: Died on day 21 to 27 of incubation; AE: Abnormal embryo; Perinatal death: Drowned in the egg; Hatched: Hatched chicklings; Σ: All incubated eggs. Pairwise comparisons (Chi-squared tests) between pairs of proportions were performed to compare the four experimental groups in every column (p < 005 was considered significant).

#### Hungarian Yellow hen × Guinea fowl male

The reverse cross carried out in year 2 used artificial insemination and resulted in a significantly less hatchlings. 98.43% of laid eggs were infertile, 1.14% have died very early (PD and BWE), 0.14% died between the days 16 and 20 (D16–20), 0.14% has perinatal death and only 0.14% (1 hybrid) hatched from 701 incubated eggs. (Table 1).

### Sperm penetration assay

Using the PSPA method (Perivitelline sperm penetration assay), 37% of the eggs of Hungarian Yellow’s hens inseminated with Guinea fowl semen, did not contain penetration holes at all. 42% of the eggs did not contain the penetration holes over the germinal disc (GD); however, the vegetative pole contained many holes which formation was different compared to the typical GD holes. 12% of the remaining eggs contained less than six holes, whereas 9% contained more than six penetration holes in GD region. These results indicate that only 9% of eggs have a chance of fertility and if there are no penetration holes, it is certain that eggs are not fertilized^65^.

### Karyotype analysis of hybrids and cytogenetic analysis of dead embryos

**Figure 4:**
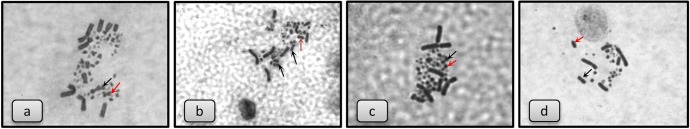
Karyotypes of hybrids and abnormalities were found in dead embryos. (**a)** Karyotype of hybrid female with ZW chromosomes and 5. metacentric chromosome from GF. (**b)** Karyotype of hybrid male with ZZ chromosomes and 5. metacentric chromosome from GF. (**c)** Aneuploid hybrid karyotype (2n-1) from a dead embryo. Absence one chromosome from the 1. pair. (**d)** Haploid karyotype from a dead embryo shows haploid/diploid mosaicism. (Z chromosome indicated with black arrow, 5. chromosome indicated with red arrow).

The expected karyotype of the hybrids is based on the *Galliformes* karyotypes described by Shibusawa *et al*.^66^. Accordingly, the hybrid karyotype consists two large submetacentric, two acrocentric, and two telocentric pairs, to which two sex and the micro chromosomes join. The size of the metacentric chromosomes of 5 pairs of Guinea fowl chromosomes is very similar to the chromosome Z (Fig. 4). There are no metacentric chromosome pairs beside the sex chromosomes of the domestic fowl. Thus, in metaphase spreads of female hybrids a smaller and a larger metacentric chromosome (Fig. 4a) is present, while a smaller and two larger metacentric chromosomes are visible in males (Fig. 4b).

Chromosomal analyses of 191 samples were performed. Based on these results, the proportion of males was 56.76%, and the females were 43.24%. According the chromosome analysis, 2.7% of the samples contained detectable chromosomal abnormalities, which is similar to other published results^67^. Two types of chromosomal abnormalities were observed during the investigations: aneuploidy and mosaicism. The ratio of specimens with aneuploid chromosome abnormality was 0.90% (Fig. 4c). The proportion of haploid/diploid (1n/2n) mosaic karyotypes was 1.80% (Fig. 4d).

### Verifying hybrid offspring using molecular genetic markers

**Figure 5:**
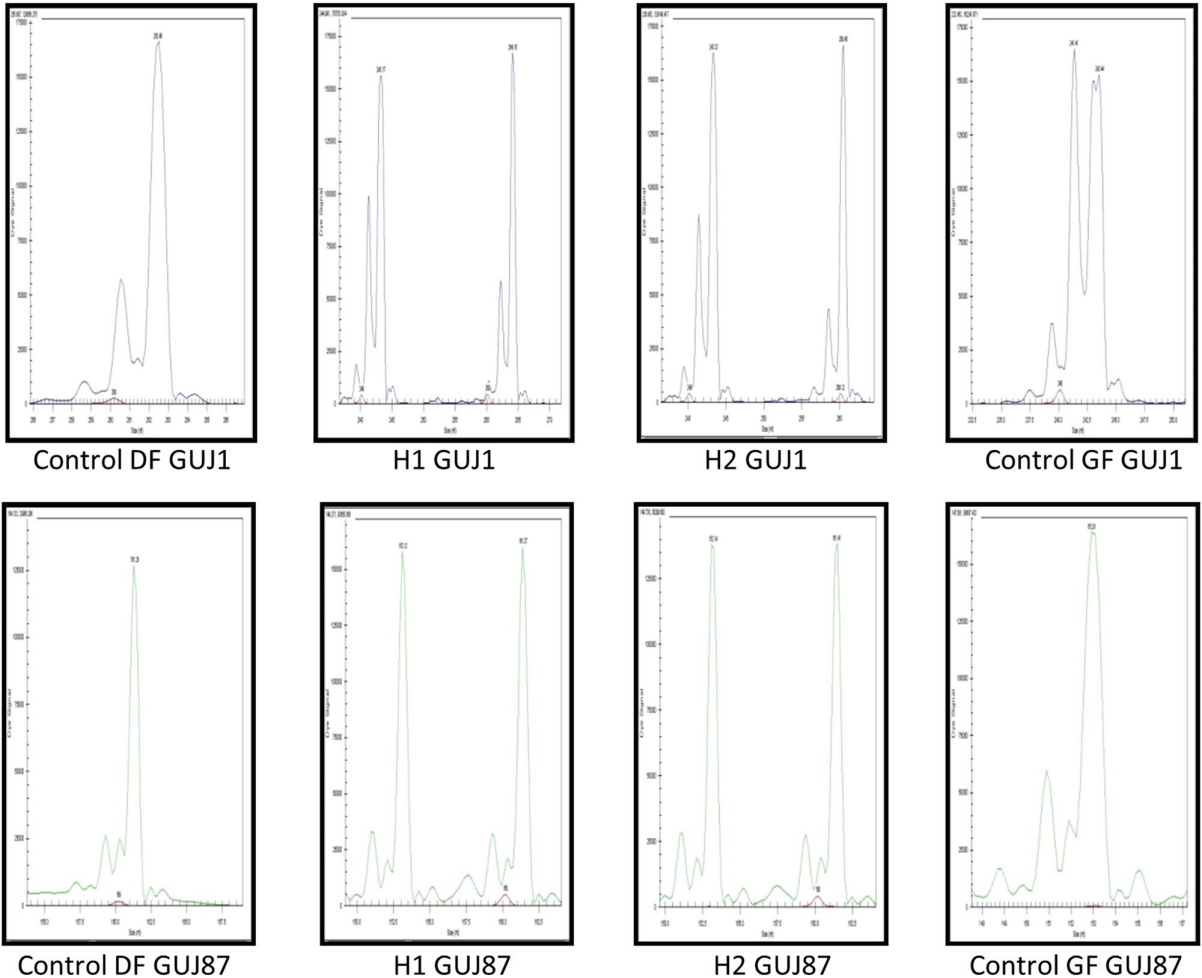
Allele sizes of hybrid, control domestic fowl and guinea fowl samples (Suppl. Fig. 1–8). Marker GUJ1 resulted allele 262 bp in domestic fowl (DF) and allele 241 bp; 243 bp in guinea fowl (GF). Hybrid 1 (H1) received one allele from domestic fowl (264 bp) and another from guinea fowl (243 bp). Hybrid 2 (H2) also have one allele from chicken (260 bp) and another from guinea fowl (243 bp). In case of marker GUJ87 there is allele 161 bp in domestic fowl (DF) and allele 153 bp in guinea fowl (GF) in homozygous form. Both hybrids (H1, H2) received both alleles (153 bp and 161 bp).

In case of the marker GUJ1: 260 bp, 262 bp and 264 bp allele sizes were observed in control chickens, while 241 bp and 243 bp allele sizes were detected in control Guinea fowl. In individuals which appeared to be hybrids, 260 bp or 264 bp and 243 bp size alleles were found. For marker GUJ87, the amplified allele sizes were 161 bp in chicken and 153 bp in Guinea fowl. Both alleles were detected in the putative hybrid individuals (Fig. 5). Based on the microsatellite marker analysis, all of the 38 putative hybrid individuals were hybrids.

### Immunostaining of PGCs of the hybrid embryos

**Figure 6:**
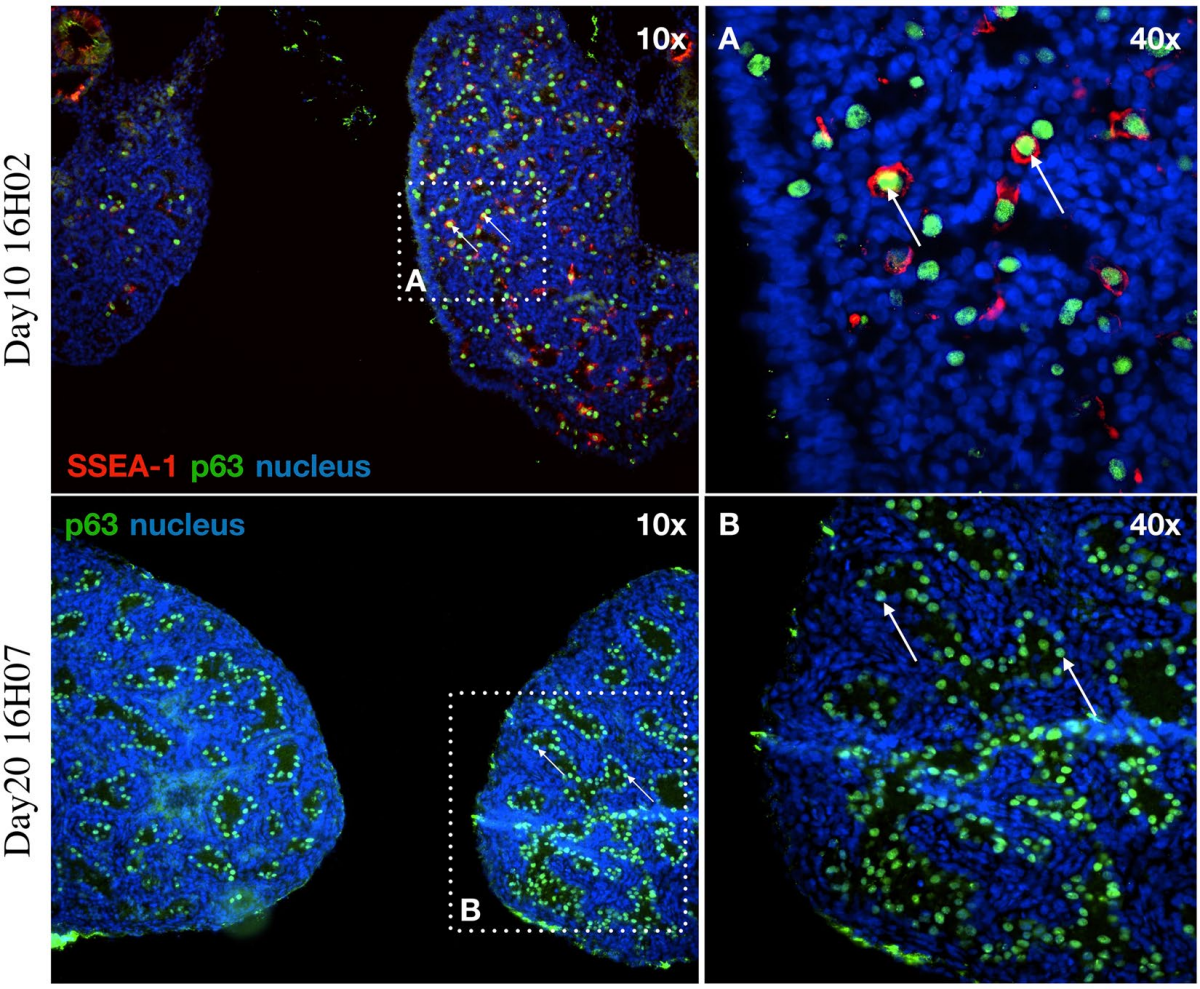
Examination the gonads of the hybrid embryos: p63 and SSEA-1 immunostaining identify the endogenous PGCs in the gonads of hybrid embryo at day-10 (16H02) and p63 at day-20 (16H07). (**A)** The SSEA-1 expressing PGCs are red colored on the cell surface. The p63 expressing PGCs are green colored in nucleus. White square shows the cells on the picture (**A)** (right top). (**B**) The p63 expressing PGCs are green colored in nucleus. White square shows the cells on the picture (**B)** (right bottom). White arrows demonstrate two host derived PGCs. For nuclear staining (nucleus) we used Hoechst 33342 staining (blue).

**Figure 7:**
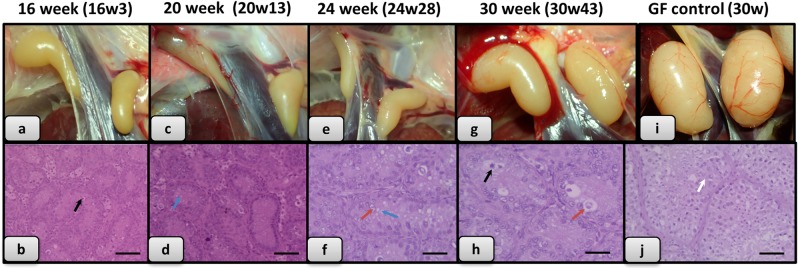
Histological analysis of gonads of the raised hybrids. (**a)** Gonads of 16 week old hybrid (Individual no. 3); (**b)** Histological section of this gonad (No. 3); (**c)** Gonads of 20 week old hybrid (No. 13); (**d)** Histological section of this gonad (No. 13); (**e)** Gonads of 24 week old hybrid (No. 28); (**f)** Histological section of this gonad (No. 28); (**g)** Gonads of 30 week old hybrid (individual no. 43); (**h)** Histological section of this gonad (No. 43); (**i)** Gonads of 30 week old Guinea fowl control; (**j)** Histological section of this gonad. (Degenerate cells with pyknotic nucleus separated from the interstitium indicated with black arrow, foamy cytoplasm indicated with blue arrow, foamy nucleus, loosened chromatin structure indicated with red arrow; spermatozoa indicated with white arrow. Scale bar b, d and j: 50 μm; f and h: 25 μm).

After immunostaining with germ cell specific marker p63^68^, PGCs were observed in each sample of gonads of the three 10-day-old (Fig. 6), one 18.5-day-old, four 20-day-old embryos (Fig. 6) and one adult hybrid individual (Suppl. Fig. 10). It is also interesting to note that out of the eight D10, D18.5 and D20 embryonic samples examined, four were females based on gonadal structure (Fig. 6A). In contrast, the 38 raised hybrids all were males (Fig. 7). At the female samples (Fig. 6A) many germ cells show co-staining of p63 and SSEA1. The germ cells are distributed throughout the gonad, not concentrated in the cortex. The p63 positive cells are circularly located along the seminal vesicles in male samples. (Fig. 6B).

### Male hybrid offspring are sterile

Male Yellow Hungarian chickens reach sexual maturity at 22 weeks. Male Guinea fowl reach sexual maturity at 24 weeks. Histological sections of gonads from 16 weeks to 20 weeks old hybrids were examined and tubular structures of irregular diameter with 2–3 cell layers were observed, minimum proliferation activity was observed. Signs of sperm formation, spermatocytes were not found. Inactive or infantile testicular cells could be observed. (Fig. 7).

From week 22 to week 30, the hybrid testes samples displayed a typically normal tubular structure. However the cells are polymorphic. Sporadically giant cells are visible. In the lumen of the tubules some epithelial cells with foamy nucleus structures or pericromasis are observed. The germinal epithelium is not active; signs of spermiocytomorphogenesis were not visible (Fig. 7).

Generally it can be assumed that the examined 38 histological samples all originated from male individuals. The normal tubular structure suggests that the gonads of the hybrids may be suitable for hosting of donor PG cells and to produce gametes.

### Injection of GFP labeled PGC lines into the 3 days old hybrid embryos

**Figure 8:**
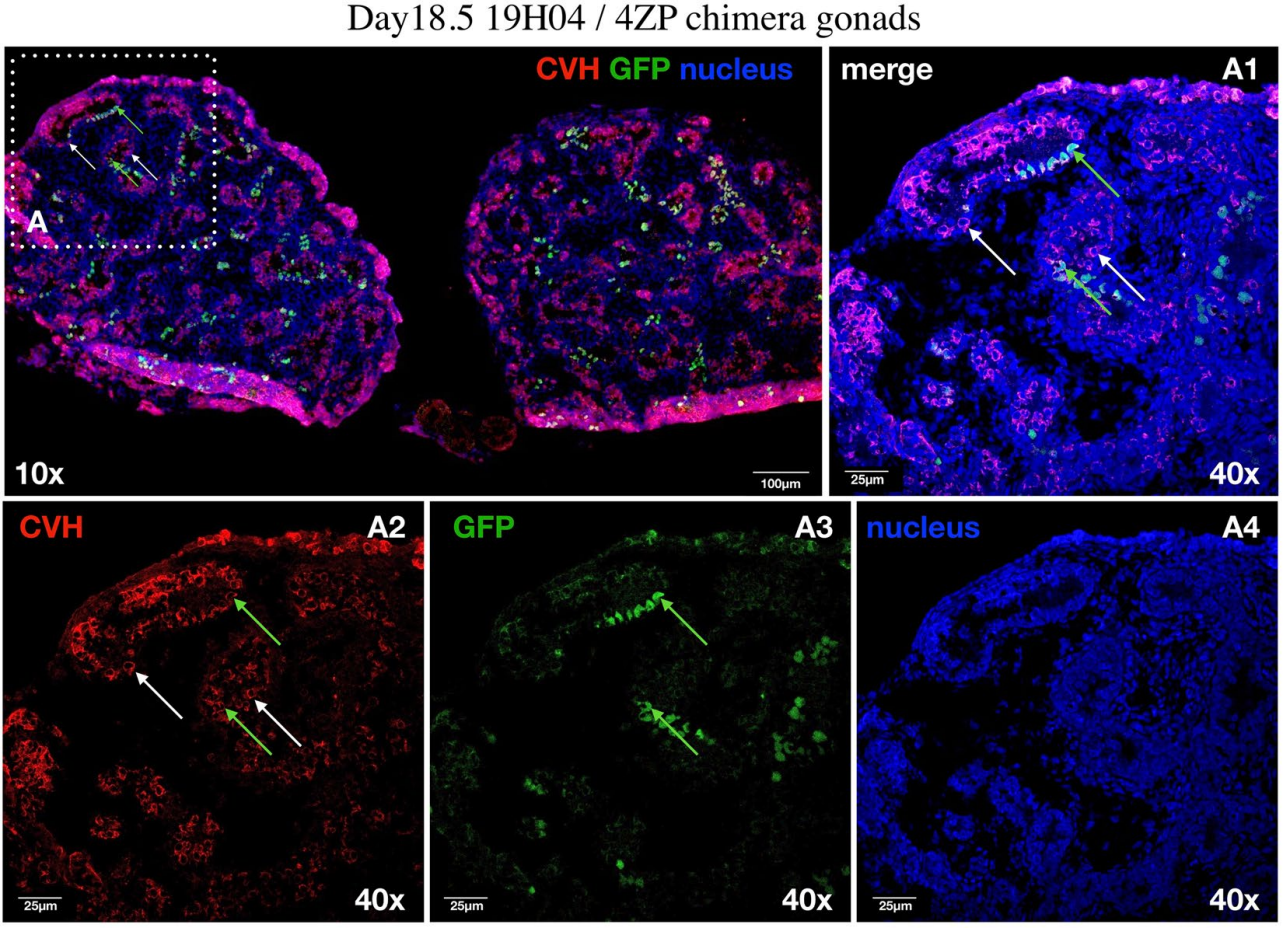
Identification of the endogenous host-derived and injected GFP expressing donor derived PGCs in the left and right gonads of 18.5-day-old hybrid embryo (19H04). (**A)** CVH expressing PGCs are red colored in the cytoplasm. The donor derived GFP expressing PGCs are green colored. White square shows the cells on the picture (**A1–A4)**. Whyte arrows demonstrate two host derived PGCs. Green arrows indicate two integrated donor derived GFP expressing PGCs. (**A1)** Confocal merge images of CVH (red), GFP (green) and nuclear stained (blue) PGCs. (**A2)** Confocal images of CVH (red) stained PGCs. (**A3)** Confocal images of GFP expressing PGCs. (**A4)** Confocal images of nuclear stained PGCs. For nuclear staining (nucleus) we used TO-PRO®-3 stain (blue). Scale bars: 100 μm (**A**), 25 μm (**A1–A4**).

In total, 147 recipient hybrid embryos were injected with GFP expressing 4ZP PGCs. Incubated eggs were analyzed at day 7.5 (30 eggs), day 14.5 (75 eggs) and day 18.5 (42 eggs). From the 15 living 7.5-day embryos 12 contained GFP positive PGCs (H1, H3, H6, H7, H8, H10, 19H12,19H14, 19H17, 19H19, 19H23, 19H25; Suppl. Fig. 11). All of 14.5- day-old embryos (**n** = **4**; H17, H25, 19H89, 19H90) and 18.5-day old embryo (**n** = **1**; 19H04) contained GFP positive cells in the gonad (Fig. 8) (Table 2).

**Table 2:** Developmental rate of 3-day-old hybrid recipient embryos injected with 4ZP PGCs.

Age of embryos	No. injected embryos	No. live embryos	No. GFP-positive gonads
7.5-day	30	15 (50%)	12 (40%)
14.5-day	75	4 (5.3%)	4 (5.3%)
18.5-day	42	1 (2.4%)	1 (2.4%)
Total No.	147	20 (13.6%)	17 (11.6%)

## Discussion

Investigations have been published since the 1930s concerning Guinea fowl hybrids^39,48,50^. A large number of publications focused on phenotype, behavior or possible fertility problems, we have not found any study analyzing the karyotype and genetic status of hybrids, or explored the possible causes of the fertility problems.

Van de Lavoir *et al*.^69^ used Guinea fowl as a xenogeneic recipient of chicken PGC lines. The GFP-expressing chicken PGCs were incorporated into the Guinea fowl gonads and colonized them. Chicken hens were inseminated with this dual-origin sperm. Most of the offspring were product of the union of chicken eggs with chicken sperm express either GFP or naked neck trait and only six were hybrids. This investigation showed that is not necessary for the PGCs, that the recipient belongs to the same species.

Our investigations focused on the suitability of the sterile hybrid offspring for genome preservation research as universal recipients. According to our results, Guinea fowl hen inseminated with sperm originated from the domestic cockerel can provide enough fertile eggs for injection of the donor PGCs using the 3 day hybrid embryos as recipients. The resulting hybrids are sterile and produce sperm only from the donor.

According to Shibusawa *et al*.^66^, the fifth chromosome of the two parent lines has different forms. According to Mott *et al*.^28^ due to the differences in morphology the synapsis of homologous chromosomes from the parent species does not occur. Therefore, the gametogenesis stops after the first stage of meiosis which causes the sterility of hybrids. This was confirmed by our histological and by immunohistochemical analysis on hybrid gonads. Histological analysis showed that hybrids do not produce spermatocytes their own gametes, but the structure of the seminiferous tubules may be suitable for the production of gametes developing from donor PGCs. According to our immunohistochemical studies, the hybrids have own germ cells but these do not develop to sperm cells during sexual maturation (Suppl. Fig. 9-10). This was also supported by injection of GFP labeled PGCs. The major issue encountered was that we obtained only male hybrid hatchlings from our matings. Therefore, further investigations are needed to determine whether female (ZW) PGCs are able to colonize the gonad of male (ZZ) hybrid recipients, integrate into the gonad, and efficiently form viable spermatozoa from a ZW genotype. According to Naito *et al*.^70^ production of donor-derived offspring was successful even when opposite-sex donor PGCs were injected into the recipients. Donor-derived hatchlings were produced from male chimeric chickens 4 out of 18 times (22.2%) and 2 out of 18 in case of female chimeric chickens (11.1%). However, the rates of donor-derived offspring from the chimeric chickens were 0.4–0.9% in male donor and female recipient and 0.1–0.3% in female donor and male recipient^70^. Liu *et al*.^71^ also reported that oogenesis of chicken primordial germ cells (ZZ) resulted in uniparental offspring. In this experiment the developmental plasticity of *in vitro* cultured PGCs (ZZ) to differentiate into functional ova in the ovary of germline chimeric chicken host was proved. Donor PGC-derived uniparental chicken offspring was hatched. These results demonstrate that avian PGCs can differentiate also into functional germ cells in a gonad of the opposite sex. Taking all these into consideration, the male hybrids might be a suitable host for PGCs from both sexes and produce functional gametes.

Furthermore, the sterility and inviability of the male hybrids produced in our study follows Haldane’s rule^72^, which states that “when in the F1 offspring of two different animal races one sex is absent, rare or sterile, that sex is the heterozygous (or heterogametic) sex”. In the case of Guinea fowl – domestic fowl hybrids, an extreme case of the Haldane’s rule is manifested, when the female sex (ZW) is completely absent.

One surprising result from our study was the observation that if the domestic chicken female (hen) was inseminated with Guinea fowl semen the proportion of unfertile eggs increased dramatically. For this reason a sperm penetration assay was performed. According to earlier studies using pure bred chickens, the number of penetration holes located above the germinal disc can vary from 0 up to 1000, although the presence of six holes indicates that the egg may be fertilized. If there are fewer than six holes, there is little chance of fertility and if there are no penetration holes, it is certain that eggs are not fertilized^65^. Based on our results, we assume that only a limited amount of the spermatozoa are able to reach the site of fertilization but the appearance of the penetration holes are not as typical as in the chicken eggs. The poor fertility data can be explained by several reasons: the suspected chemotaxis, the activity of sperm binding receptors and/or the induction of acrosome reaction which all help spermatozoa to find the germinal disc and create the fusion between gametes does not work properly in the case of this interspecific hybrid. If fertilization still occurs, this hybrid does not differ in anything from the result of the reciprocal crossing.

## Conclusions

Crossing Guinea fowl with domestic fowl was successful but only with female Guinea fowl crossed with male chicken. In the case of the reverse crossing, 98.4% of the eggs were infertile. Based on the investigations of this study, the observed offspring from the successful crossing were sterile male hybrids. These sterile hybrids have endogenous germ cells, but they do not develop into sperm cells. Accordingly, an extreme form of Haldane’s rule was manifested. In the case of reversed crossing, the inappropriate chemotaxis was the presumed reason of the high rate of infertile eggs. Spermatozoa penetrations could not located in the vitelline membrane over the surface of the blastoderm. The sterile males produced in this study might be suitable recipients for male chicken PGCs and possibly for female PGCs. This research work shows that the sterility of hybrids can be used in gene conservation to be a universal host for PGCs of different avian species.

## Supplementary information

Supplementary information for Investigation of the Guinea fowl and domestic fowl hybrids as potential surrogate hosts for avian cryopreservation programmes

## Data Availability

The datasets used and analyzed during the current study are available from the corresponding author on reasonable request.
