# Supplementary material for: Investigation of the Guinea fowl and domestic fowl hybrids as potential surrogate hosts for avian cryopreservation programmes
Source: Sci Rep. 2019 Oct 3;9:14284. doi: 10.1038/s41598-019-50763-3 (PMC6776557; doi:10.1038/s41598-019-50763-3)
Supplement: Supplementary file 1 — Supplementary information for Investigation of the Guinea fowl and domestic fowl hybrids as potential surrogate hosts for avian cryopreservation programmes [file 41598_2019_50763_MOESM1_ESM.pdf]

Supplementary information

for

Investigation of the Guinea fowl and domestic fowl hybrids as potential surrogate hosts for avian cryopreservation programmes

Mariann Molnár[1], Bence Lázár[1,2], Nikoletta Sztán[1], Barbara Végi[1], Árpád Drobnyák[1], Roland Tóth[2], Krisztina Liptói[1], Miklós Marosán[3], Elen Gócza[2], Sunil Nandi[4], Michael J. McGrew[4], Eszter Patakiné Várkonyi[1*]

Supplementary figures 1-8.: Allele sizes of hybrid, control domestic fowl and guinea fowl samples

Marker GUJ1 resulted allele 262 bp in domestic fowl (DF) and allele 241 bp; 243 bp in guinea fowl (GF). Hybrid 1 (H1) received one allele from domestic fowl (264 bp) and another from guinea fowl (243 bp). Hybrid 2 (H2) also have one allele from chicken (260 bp) and another from guinea fowl (243 bp). In case of marker GUJ87 there is allele 161 bp in domestic fowl (DF) and allele 153 bp in guinea fowl (DF) in homozygous form. Both hybrids (H1, H2) received both alleles (153 bp and 161 bp).

Figure 1: GUJ1_DF

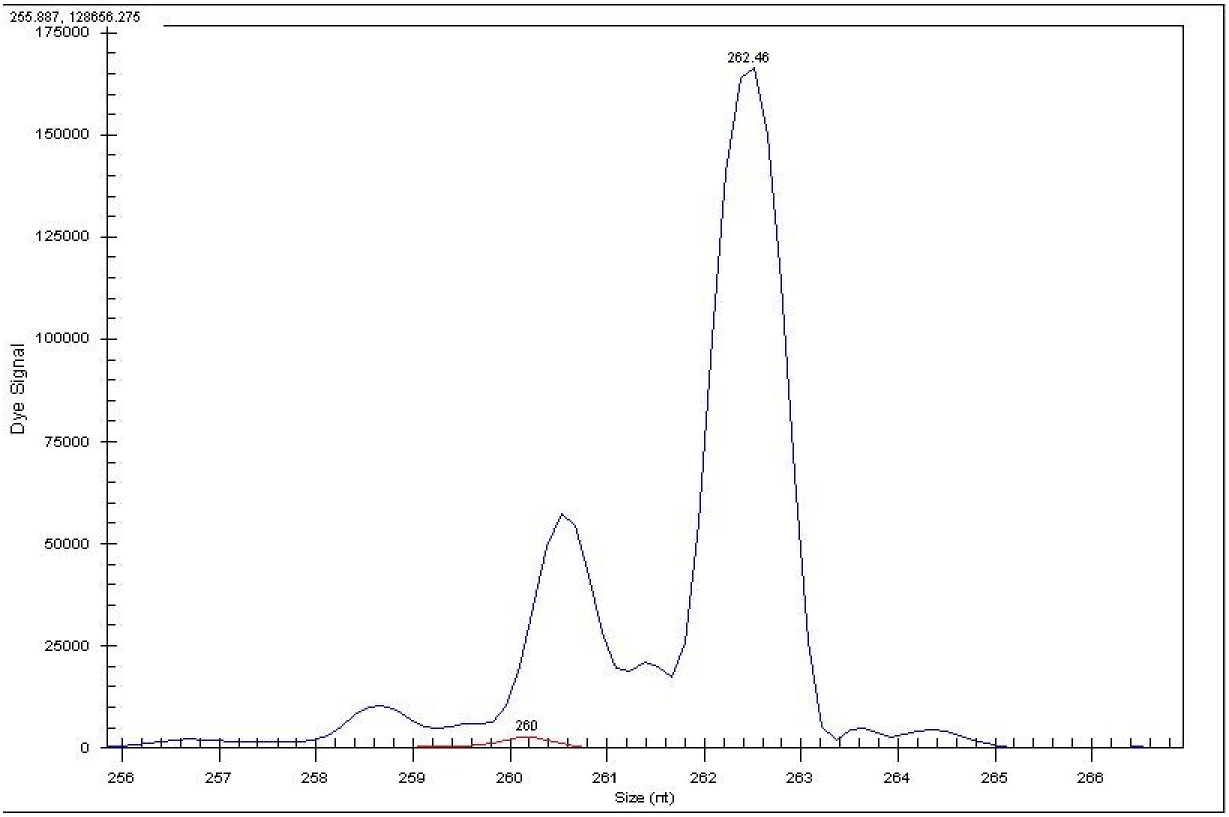

Figure 2: GUJ1_GF

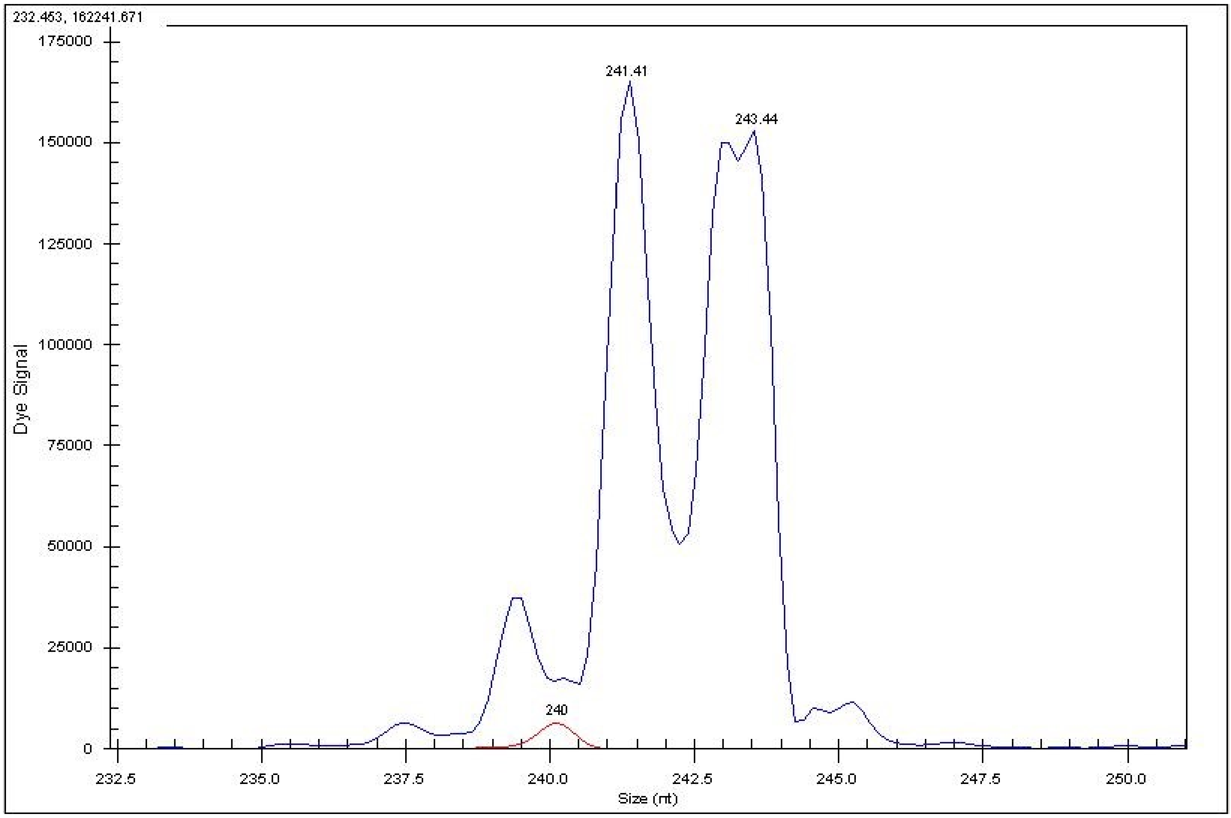

Figure 3: GUJ1_H1

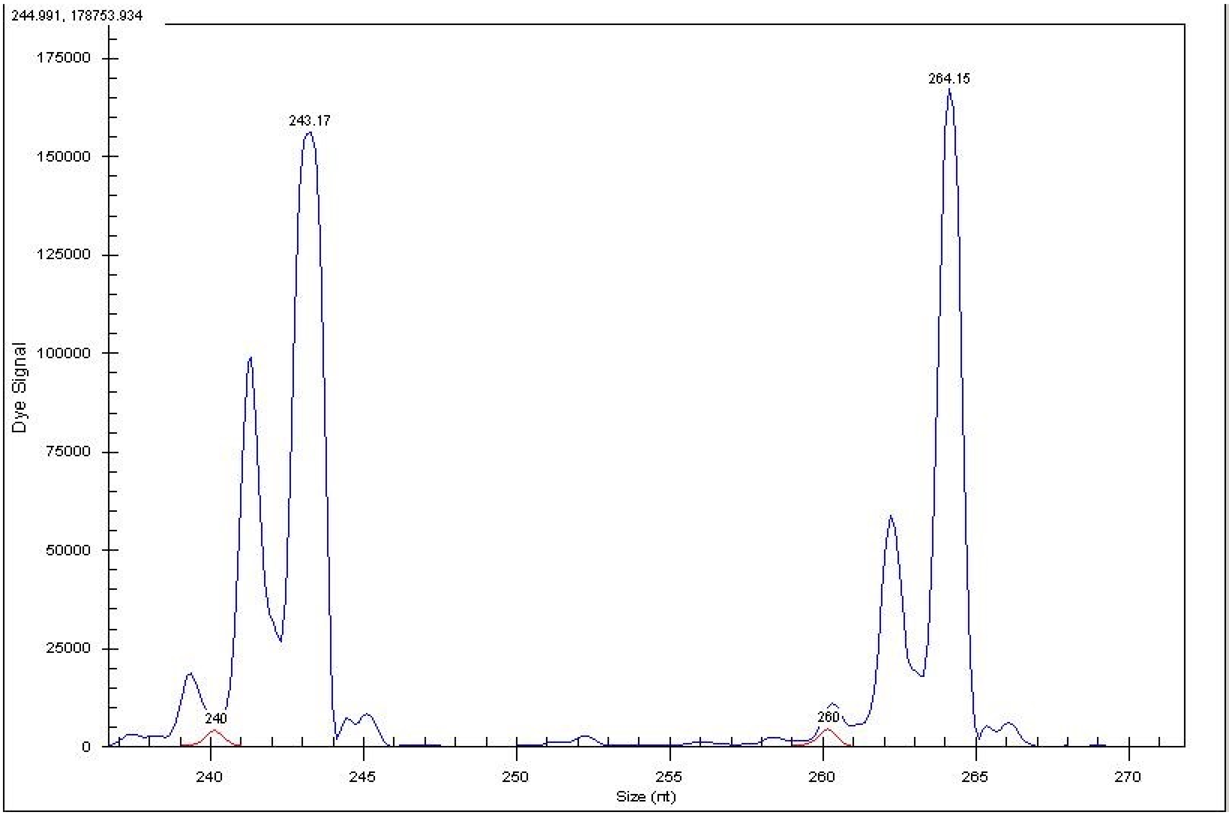

Figure 4: GUJ1_H2

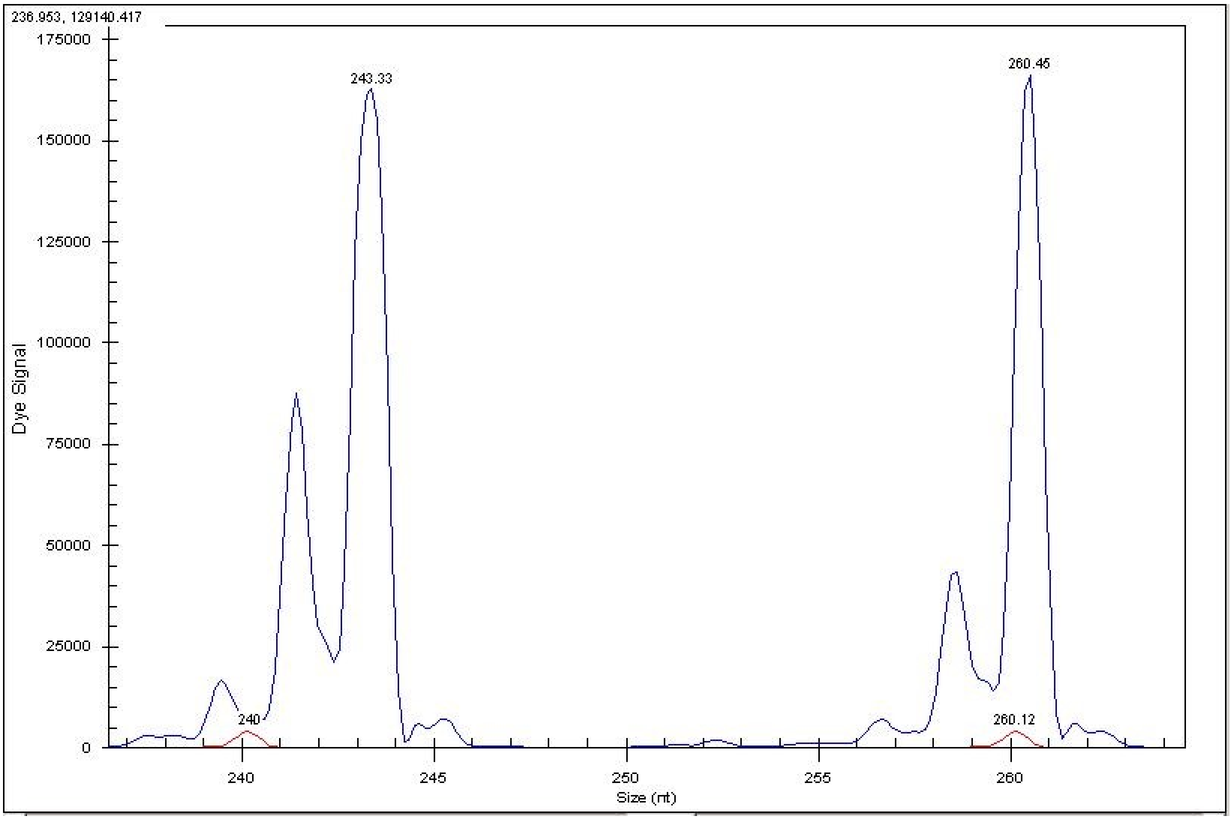

Figure 5: GUJ87_DF

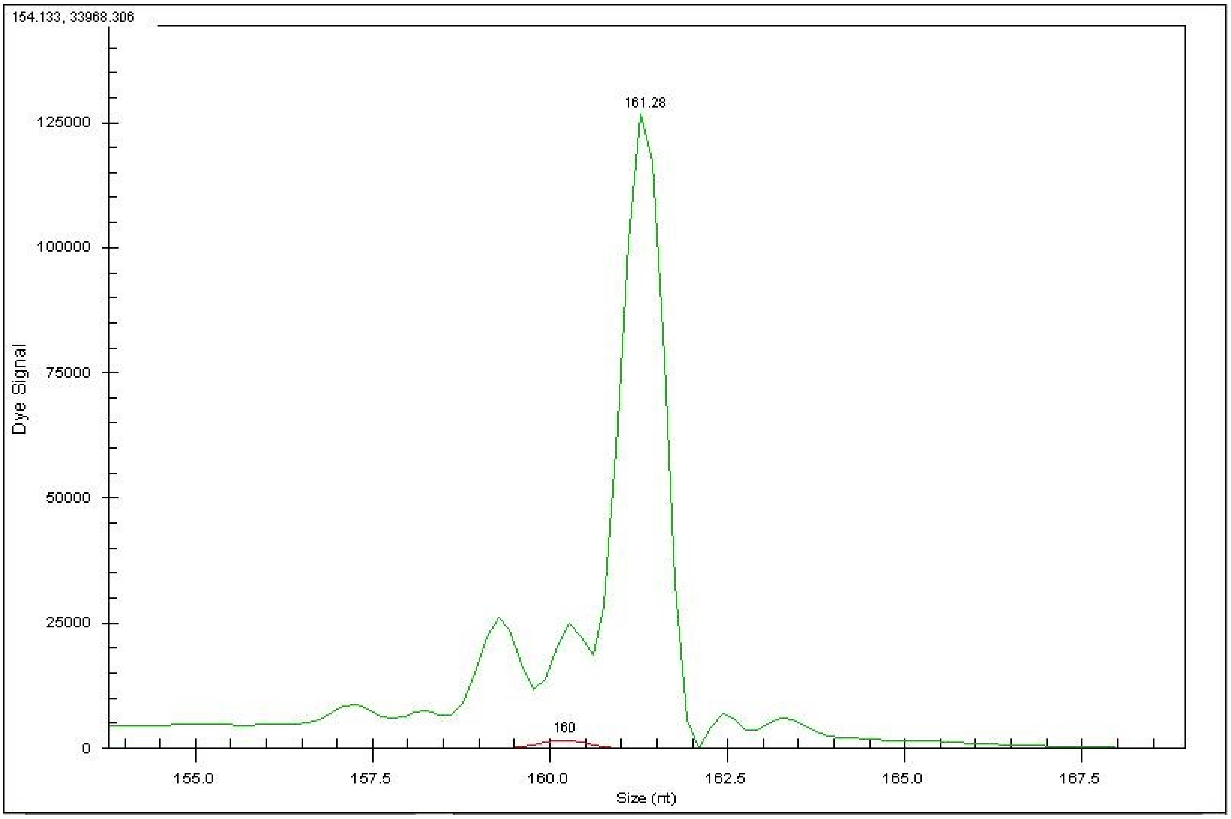

Figure 6: GUJ87_GF

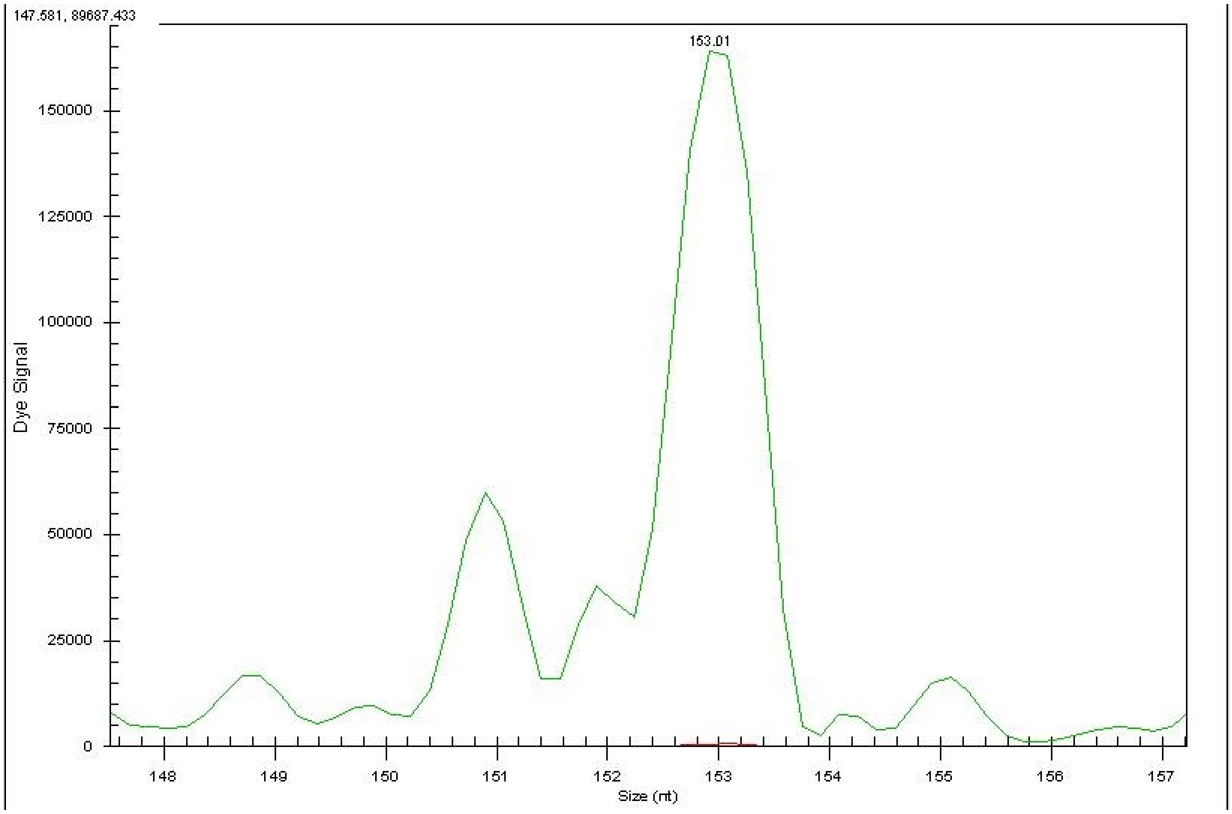

Figure 7: GUJ87_H1

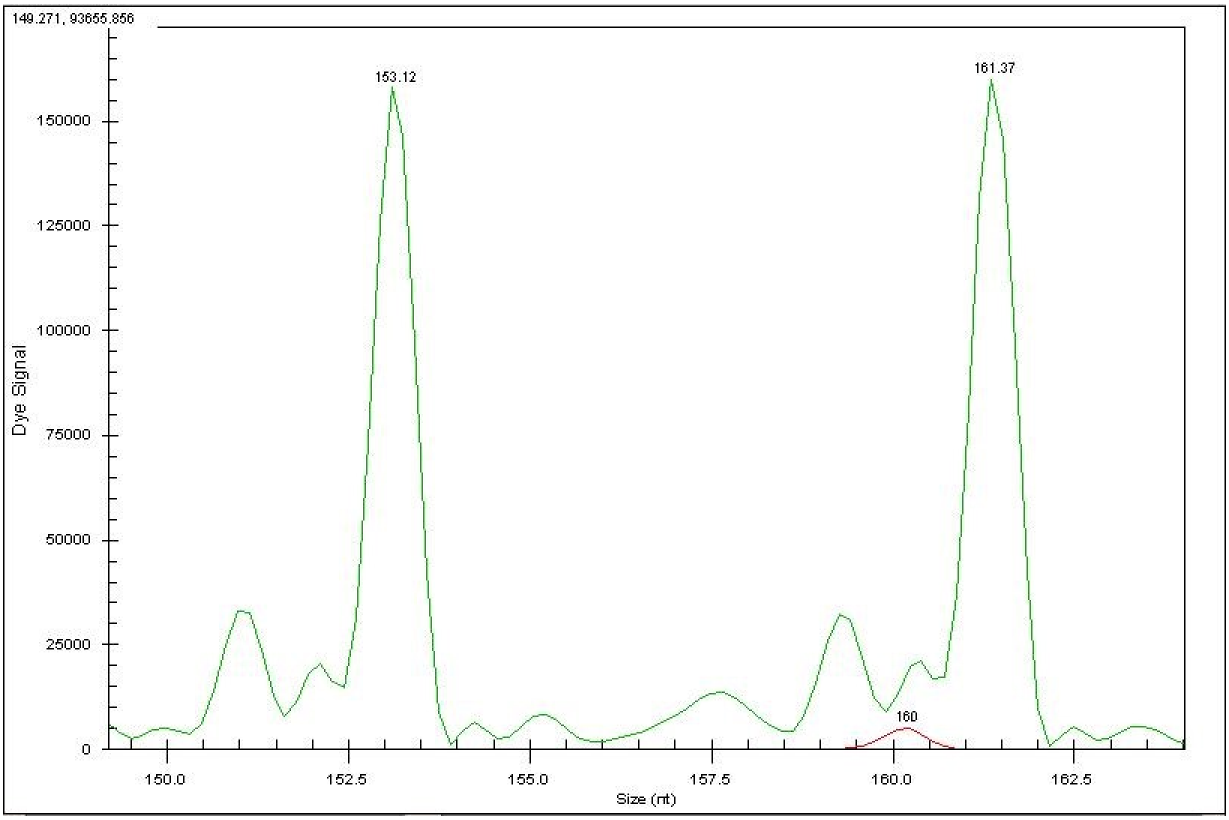

Figure 8: GUJ87_H2

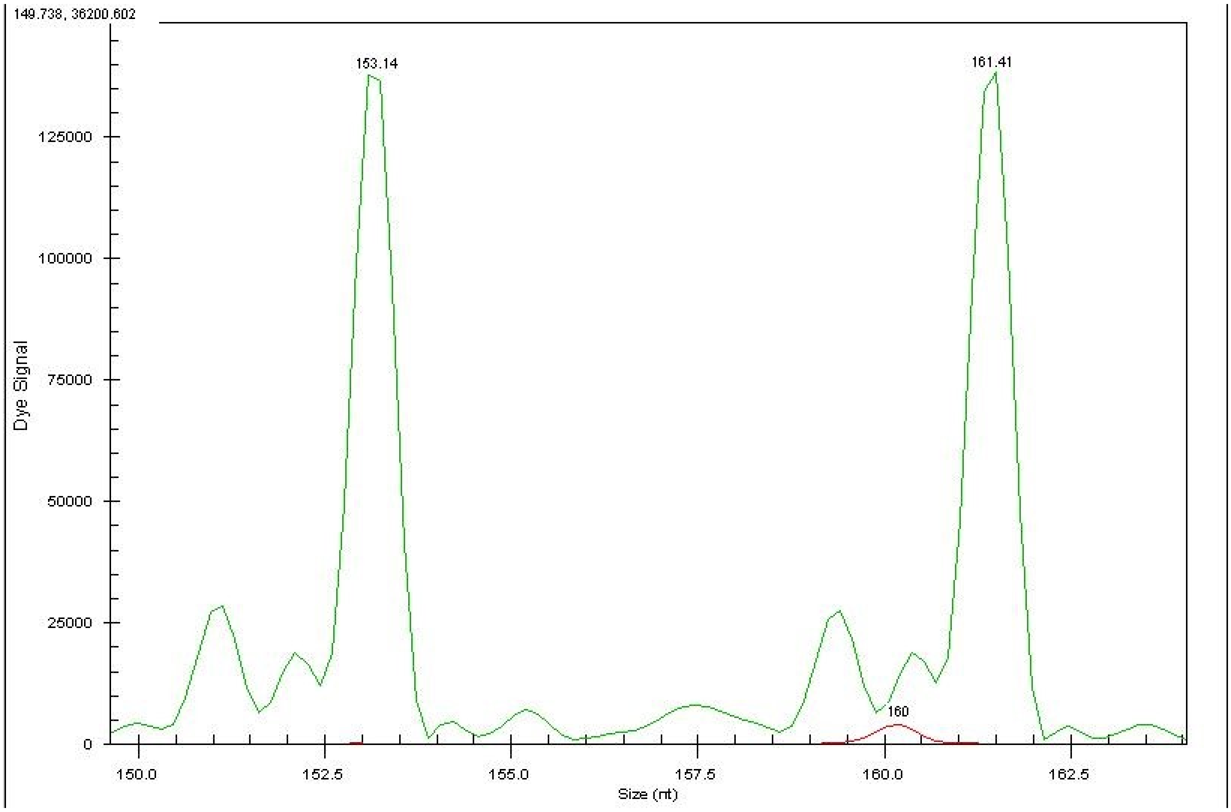

Figure 9: Examination the gonads of an adult hybrid and adult cockerel control : p63 and DAZL immunostaining. Gonad of adult Hungarian yellow cockerel control.

DAZL

P63

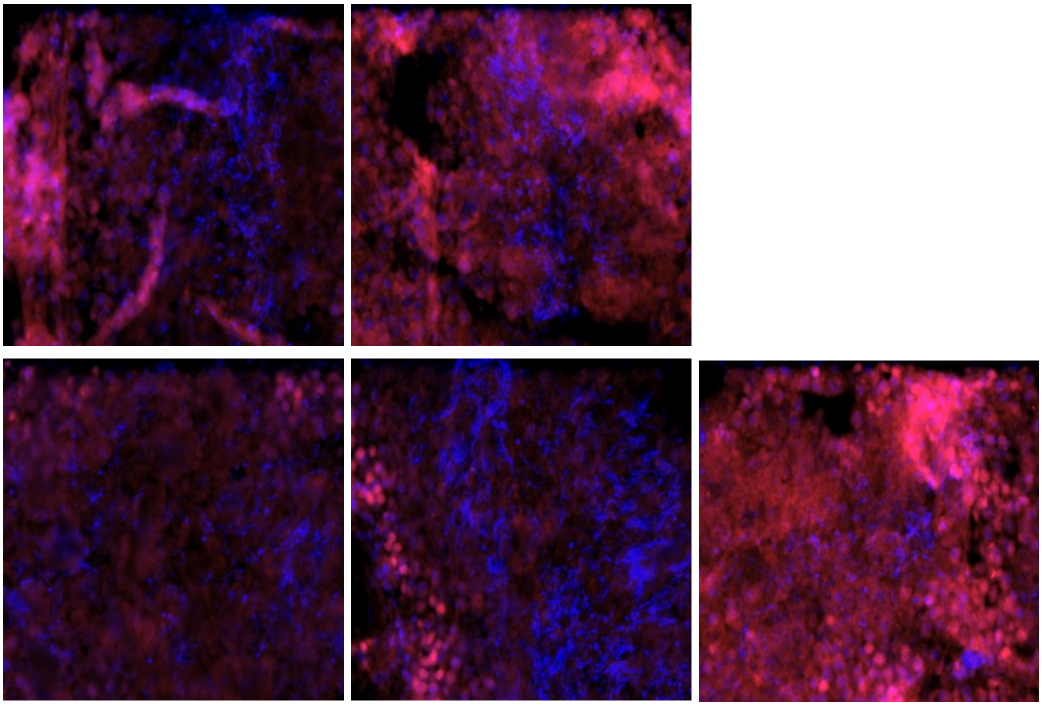

Germ cells and sperm cells are also visible

Figure 10: Examination the gonads of an adult hybrid and adult cockerel control : p63 and DAZL immunostaining. Gonad of an adult hybrid.

DAZL

P63

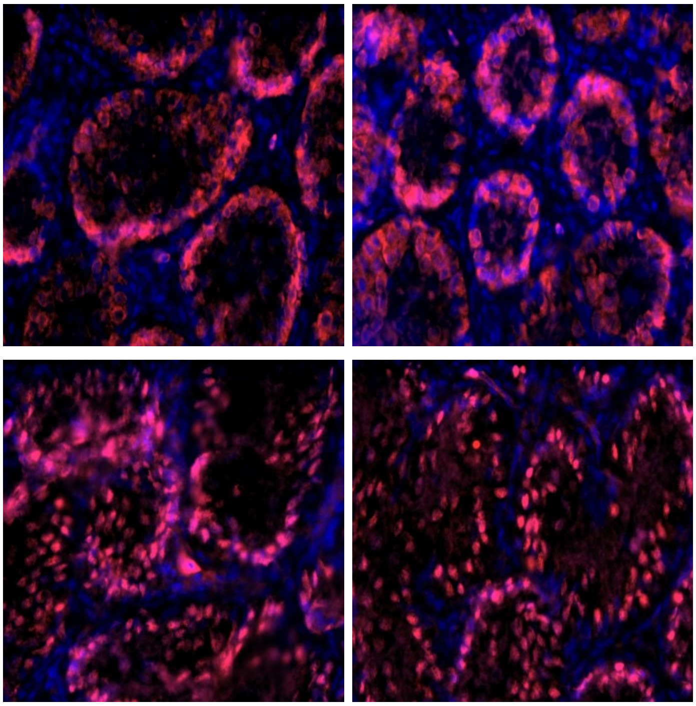

Hybrid's gonad looks like the immature testis.

Figure 11: GFP expressing PG cells in the gonad of hybrid recipient embryos injected with 4ZP PGCs.

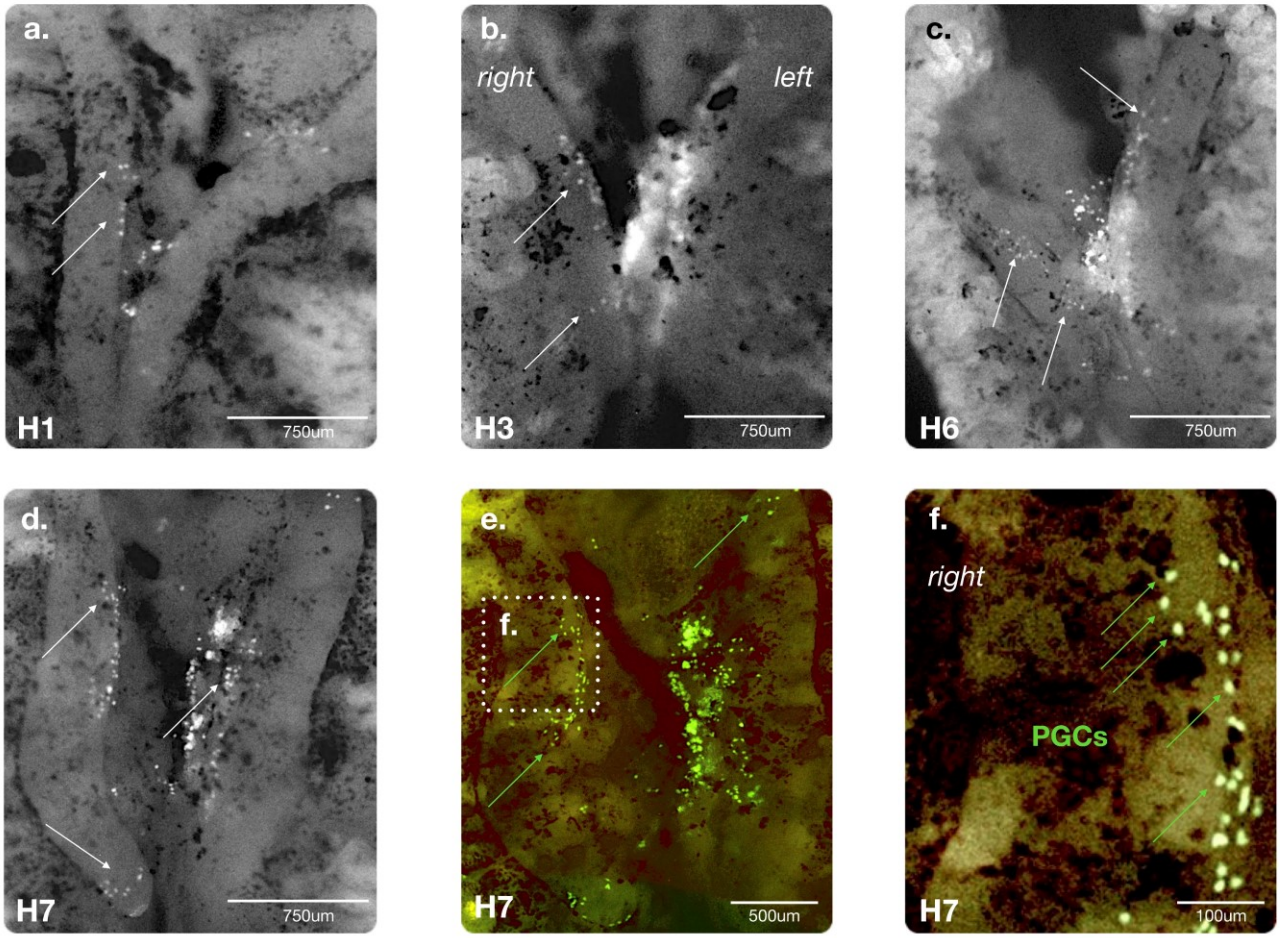

Gonads of 7.5-day-old hybrid recipient embryos (H1, H3, H6, H7). The GFP expressing PGCs entered and colonized the gonads indicating that the hybrid animals might be suitable universal recipients. Arrows indicate the integrated GFP expressing 4ZP cells in the gonads of hybrid embryos. PG cells are visible in all embryonic gonads both in the right and the left gonads.

a.: Gonads of H1 hybrid (scale bar: 750 µm); b.: Gonads of H3 hybrid (scale bar: 750 µm); c.: Gonads of H6 hybrid (scale bar: 750 µm); d.: Gonads of H7 hybrid (scale bar: 750 µm); e.: Gonads of H7 hybrid, green arrows indicate the integrated GFP-expressing PGCs into the gonads (scale bar: 500 µm); f.: Right gonad of the H7 hybrid (scale bar: 100 µm)

a., b., c., d: bright field images; e., f: fluorescent images
